# Sexual and reproductive health knowledge and behavior among adolescents living with HIV in Zambia: a case study

**DOI:** 10.11604/pamj.2017.26.71.11312

**Published:** 2017-02-20

**Authors:** Therese Ntigwa Ndongmo, Clement Bertin Ndongmo, Charles Michelo

**Affiliations:** 1 Department of Public Health, School of Medicine, University of Zambia, Lusaka, Zambia; 2 Department of Biomedical Sciences, School of Medicine, University of Zambia, Lusaka, Zambia

**Keywords:** HIV, adolescents, sexual reproductive health, knowledge and behavior, Zambia

## Abstract

**Introduction:**

As HIV infected adolescents mature into adulthood, they are confronted with issues related to sexuality and sexual reproductive health (SRH). An estimated 68,000 adolescents aged 10-19 years are living with HIV in Zambia. The current study explores their sexuality and SRH experience and needs.

**Methods:**

This was a mixed method analytical cross-sectional study. Adolescents at a tertiary hospital were surveyed on their sexuality and SRH experiences. Bivariate analyses on SPSS were used to assess factors associated with selected behaviors. Emerging themes from open-ended questions qualitative data were explored using content analysis.

**Results:**

A total of 148 adolescents (63.5% females) aged 15-19 years were surveyed. Majority (77.0%) had secondary education; 77.2% currently in school; 40.1 % had a boy or girlfriend; 15.1% have ever had sex, of whom only 61.1 % reported consistent condom use. About 68.9 % expressed intention to have children; 2.1% of girls had been pregnant before. Of 52 respondents, 19.2% had a sexually transmitted infection (STI) before. Not being in school was a significant predictor, for knowing where to access information about sex (OR= 2.53; 95% CI:1.10-5.82; p=0.02), and also for ever gone there (OR=2.61; 95% CI:1.04-6.58; p=0.03).

**Conclusion:**

The survey of HIV infected adolescents attending a tertiary hospital in Zambia found that their sexuality and SRH needs remain similar to those of the general adolescent population in terms of counseling in sexual matters, family planning and STI services. More efforts are needed to provide for adolescent health care needs, especially those living with HIV.

## Introduction

Approximately 36.7 (34.0–39.8) million people were living with HIV in 2015 globally [1]. Children and young people bear considerable share of this burden. In 2013, there were 3.2 (2.9-3.5) million children younger than 15 years living with HIV and 4 (3.6 –4.6) million young people 15–24 years old living with HIV, 29% of whom were adolescents aged 15–19 years [2]. In Zambia, estimated 13.3% of the population aged 15-49 years are currently living with HIV/AIDS [3]. Adolescents 10-19 years old are especially affected by the AIDS epidemic in Zambia, with about 68,000 (60,000 – 80,000) estimated to be infected by end of 2015 [1]. With increasing access to antiretroviral (ARV) treatment, many more perinatally infected children are now reaching adolescence [4–6].

Adolescence is a period of transition between childhood and adulthood characterized by physiological, emotional and sexual development; it is also a period of individual autonomy, growing sense of identity and self-esteem [7, 8]. HIV infected adolescents are prone to other factors relating to their infection, including disease progression, neuro-cognitive deficits, HIV stigma, and disclosure of infection status, combined with environmental factors such as substance use, poverty, inner-city stress, and disrupted family attachments [9, 10]. HIV-infected adolescents face additional unique complexities related to the impact of HIV on physical and mental health, and normative developmental processes such as school functioning, puberty, growth, peer relationships and sexuality [11–13]. Like with general adolescent populations [7, 14], studies show that, those living with HIV are beginning to explore their sexuality, are dating and some are becoming sexually active [15–18]. In a 2013 Zambia study, 21% of 15-19 year old adolescents living with HIV (ALHIV) reported ever had sex, with 57% having experienced forced sex (24% male and 76% female) [19]. Although the legal age for sex in Zambia is 16 [14], the 2007 Zambia Demographic Health Survey revealed that among the general population aged 15-19 years, 12.3% and 16.2% of women and men respectively had sexual debut before the age of 15 [20]. The Zambia sexual behavioral survey in 2009 reported rates of 44% in 2005 and 35% in 2009, among adolescent population [21]. With sexual activity comes the occurrence of pregnancies; in Uganda for instance, 184 and 7 pregnancies were reported in 2006 alone from the AIDS Support Organization and the Pediatric Infectious Disease Clinic respectively among 4,696 and 600 adolescents perinatally infected (10-19 years), receiving health services at these 2 institutions [22].

Also in Uganda, pregnancy among ALHIV reached rates similar to the general population [15]; while a study in Cote d’Ivoire reported increased incidence of pregnancy among HIV-infected adolescents in care, to a level observed in adult cohorts in Sub-Saharan Africa [16]. Studies show that unprotected sex remains common; one study in Tanzania reported an increase of unprotected sex among HIV positive young people [23]. With the advent of ARV therapy, a study in the US among HIV positive youth aged 13-24 showed they were more likely to have unprotected sex with a partner they knew was HIV positive [24]. HIV positive youth sexual activity during adolescence may lead to acquisition or transmission of diseases [25]; and they are at greater risk for contracting sexually transmitted infections (STIs) due to their compromised immune system. Studies have also documented that HIV infected youth engage in alcohol use and unprotected sexual intercourse [26]. While most studies on adolescent sexuality have been conducted among general adolescent populations, little is still known about the growing subpopulation of HIV infected adolescents especially in sub-Saharan Africa; and Zambia in particular. Hence the present study was conducted to further explore sexual and reproductive health behaviors and needs among HIV infected adolescents in Zambia.

## Methods

### Study setting

The study was conducted at the University Teaching Hospital (UTH) in Lusaka, Zambia. The UTH serves as the referral center for urban and peri-urban communities in Lusaka, the capital city of Zambia. Most children requiring hospitalization are sent to the inpatient admission ward. All children testing HIV positive from urban and peri-urban communities are offered enrollment in care at the pediatric center of excellence (PCOE) located within UTH or at the local district clinic, but eventually referred to UTH PCOE at some point. According to the PCOE clinic registry in June 2012, the facility had a total of 800 enrolled on antiretroviral therapy (ART) of which approximately 300 were adolescents 10 years and older; hence the selection of this site for the study.

### Sampling

Participants were selected with a purposeful sampling process. All HIV positive adolescents attending the PCOE were consecutively approached and asked to participate in the study. This method was chosen to include all subjects over three months, a time period when they would normally visit the clinic for their routine follow up; making the sample a better representation of the population at the study site. The inclusion criteria were: HIV positive adolescents aged between 15-19 years, attending the PCOE during the study period; assent and informed assent and consent provided by participants and guardians/parents. Excluded from the study were patients younger than 15 years and patients older than 19 years and those with no consent and assent provided.

### Design

This was a non-interventional cross sectional mixed methods survey among HIV positive adolescents, about their sexual behavior, beliefs, attitudes, and practices. This method was chosen to gain a broad base of knowledge about sexual and reproductive health among the participants. The topics explored were knowledge of family planning, STIs, and sexual reproductive health behaviors.

### Data collection

The questionnaire was structured and included both open and close-ended questions. The information collected included demographic characteristics as well as access to information and support for the HIV positive adolescent. The questionnaire explored the sexual behaviors and practices, HIV prevention knowledge and practices, contraceptive knowledge and use, pregnancy and childbearing experience, and health seeking behavior. The questionnaire was self-administered and the researcher or research assistant was there to answer any participant question for clarification. Each form was assigned a unique number and a master list was maintained for reference. Data was collected from September to December 2013. Completed forms were reviewed and responses were analyzed to identify common unexpected responses that respondents made and a decision taken about how to enter them. Data was entered into an access database designed according to the questionnaire form.

### Data analysis

The data analysis was carried out using the Statistical Package for Social Science (SPSS) version 17.0. Descriptive summaries and frequencies of socio-demographic characteristics (e.g. by age, sex, schooling, living arrangement, religion, and source of health information) were carried out. Factors associated with different key sexual behavior theme variables were examined with bivariate analysis using chi square; these factors included education, gender, age, religion, and relationships. Statistical significance was established at p < 0.05 and all tests were 2-tailed. Odds ratios and 95 percent confidence intervals were calculated. Qualitative data from answers to open-ended questions were analyzed using content analysis method, and emerging themes were described.

### Ethical considerations

The study commenced after approval from University of Zambia Biomedical Research Ethics Committee, and permission granted by UTH PCOE management. Consent was obtained from all adolescents aged 18-19 years and emancipated 15-17 year olds. For all non-emancipated youth aged 15-17 years, assent was obtained as well as consent from guardians/parents. For confidentiality, names weren’t collected on the form.

## Results

### Population characteristics

Overall, 148 adolescents were included in the study; 94 (63.5%) females and 54 (36.5%) males. The mean age was 17, and 88 (59.5%) were aged 15-17 years while 60 were aged 18-19. Of all participants, the majority 114 (77%) had some secondary education, while 19 (12.8%) had some tertiary, and 15 (10.1%) had no or primary education. Of the 145 respondents, 112 (77.2%) were in school while 33 (22.8%) were not; of those not in school, 4 were employed. Regarding a source of health information, 140 (94.6%) adolescents indicated at least one source, while 8 (5.4%) didn’t answer. The most frequent source of information was health education sessions at the clinic, n=69 (49.3%); followed by television, n=66 (47.1%); phone, n=28 (20.0%) (possibly U-report, a phone based information bank developed by UNICEF); radio, n=25 (17.9%); newspapers, n=23 (16.4%); community talks, n=8 (5.7%); and internet, n=8 (5.7%). On living arrangements, 5 didn’t answer. Of those who responded, only 1 was living alone, while of the remaining 142, 81 (57.0%) were living with at least one parent, while 19 (13.4%) were living with a grandparent, and 42 (29.6%) with other relative or guardian (Table 1).

**Table 1:** Socio-demographic characteristics of HIV positive adolescents aged 15-19 years (N=148)

Characteristics	N (%)	Characteristics	N (%)
**Sex**		**Living with**	
Female	94 (63.5%)	Parents	81 (56.6%)
Male	54 (36.5%	Guardians/relatives	61 (42.7%)
**Age group**		Alone	1 (0.7%)
15-17	88 (59.5%)	**Religion**	
18-19	60 (40.5%)	Protestant	79 (57.2%)
**School attendance**		Catholic	45 (32.6%)
In school	112 (77.2%)	Other faiths	12 (8.7%)
Not in school	33 (22.8%)	Muslim	2 (1.4%)
Source of Health information*		**Level of Education**	
Health education talks	69 (46.6%)	Secondary	114 (77%)
Television	66 (44.6%)	College and above	19 (12.8%)
Telephone	27 (18.2%)	No to Primary	15 (10.1%)
Radio	25 (16.9%)	**Marital status**	
Newspapers	23 (15.5%)	Single or never married	135 (94.4%)
Others	16 (10.8%)	Married or cohabiting	8 (5.6%)

**Note:** Totals do not always round to 100% because participants did not always respond to all the questions;

* some participants indicate more than one source of health information

### General health and HIV/AIDS issues

When asked about their current health, 91 (61.5%) responded very good to excellent and 57 (38.5%) responded fair to good. As to whether their health interferes with their social activities 103 (75.2%) said no and 34 (24.8%) said yes. Among those who responded yes, and stated how that was the case (n=26), the main emerging themes included 1) not feeling or being able to do what their friends do, or being allowed to go to certain places (n=6); 2) the fear and worries of being seen taking medication, and/or what people would think, or how they are treated by family and friends (n=10); and 3) feeling bad and having question about their health condition (n=5). Some of the statements were: “I can´t do what my other friends are able to do, and that makes me feel bad”; “yes it has interfered with social activities such that I haven’t even been going to school”; “I have missed school on several occasions”; “fail to relate with my family and friends that are negative”; “not allow to go to camp with friends because of fear to see you taking ARVs”; “bring up low self-esteem”.

Out of 137 respondents on their HIV infection interfering with household chores, 94 (68.6%) indicated no interference, while 28 (20.4%) said it does interfere sometimes, and 15 (11.0%) indicated a definite yes; some explanations were: feeling tired, dizzy, and being afraid of cutting oneself. Regarding number of hospitalization, of 129 respondents, 43 (33.3%) said they were hospitalized at least once, while 86 (66.7%) hadn’t been hospitalized in the past one year. Regarding treatment, out of the 146 respondents, majority 135 (92.5%) reported being on ART, while 11 (7.5%) weren’t because not yet eligible based on their CD4 count. Of respondents on ARVs, 61 (45.2%) could remember the name of at least one medication they were taking. When asked if they knew how they might have acquired HIV infection, 143 responded. Of these, 105 (73.4%) indicated mother to child transmission, 29 (20.3 %) didn’t know, 5 (3.5%) said through contaminated sharps, while 3 (2.1 %) indicated sexual abuse, and 1 (0.7%) mentioned blood transfusion. With regards to the age at which they became aware of their HIV serostatus, of the 133 respondents, the majority 83 (62.4%) said between the ages of 10 – 14; 34 (25.6%) became aware at 15 or older; and 16 (12.0 %) became aware before 10 years. Out of 140 respondents, 71 (50.7%) learnt their serostatus from a health care worker; 47 (33.6%) from their parents, 19 (13.6%) from their relatives, and 3 (2.1%) found out by themselves by hearing their parents talk, reading their files, or searching what their medication was meant for.

### Sexuality and sexual behavior

Table 2 gives detailed information on adolescent’s sexuality and sexual behavior. Overall, 86 (58.1%) of the 148 participants could identify at least one change associated with puberty, including body size changes (breasts, hips), voice change, pubic and armpit hair, menses, and sexual desires. Of the 88 (93.6%) female who responded, the majority 52 (59.1%) had their first menses between 13-14 years old, 22 (25%) when they were 15 and above, and 14 (15.9%) at 10-12. Concerning children, 104 (88.9%) out of 117 respondents indicated intention to have children in life (63 females and 41 males); of those, 41 (40.2%) would want 2 children; 29 (28.4%) would like 3; 23 (22.5%) would want 4; 7 (6.9%) would want 5; and 2 (2.0%) would like more than 5. On marital status, 145 responded, of whom 135 (93.1%) were single or never married. Regarding friendships, of the 137 (92.6%) who responded, 55 (40.1%) admitted to having a boy or girl friend. Overall, 21 (15.1%) out of 139 respondents said they have ever had sex (12 females and 9 males); 15 had it with boy/girlfriend or spouse, while 5 indicated being forced, and 1 didn’t specify. The age at first sex was 15 years and over for 18 (85.7%) of the sexually experienced, and below 15 years for 3 (14.3%). As to how many partners they had sex with in the last 6 months, 8 said none, 10 said one and the remaining 3 said they had sex with 2 partners. Of all 21 with sexual experience, only 13 (61.9%) used condoms during the last sexual intercourse. Of 19 respondents who have disclosed their HIV status to their partner, 9 (47.3%) reported there was no negative reaction. Only 8 (42.1%) of 19 respondents knew whether their partner has been tested also. On condom use overall, 11 (61.1%) out of 18 reported using it always, 4 (22.2%) use sometimes, and 3 (16.7%) never use. Out of the 12 female adolescents who ever had sex, 2 have been pregnant (age 16 and 18) and were pregnant before being aware of their HIV status. For one the pregnancy was intended with partner consent. One respondent was pregnant, and due in six months.

**Table 2:** Sexuality and sexual behaviors among HIV positive adolescents aged 15-19 years respondents (N = 16 to 139)

Sexuality and sexual behaviors	Male	Female	Both	Respondents	%
Have boy/girl friend	17	38	55	137	40.1
Expressed intention to have children	41	63	104	117	88.9
Intended number of children (2-4)	33	60	93	117	79.5
Ever had sex*	9	12	21	139	15.1
Have informed partner about HIV status	3	6	9	19	47.4
Know if partner has been tested or not	3	5	8	19	42.1
*Condom use during sex*					
Always	6	5	11	18	61.1
Sometimes	1	3	4	18	22.2
Never	1	2	3	18	16.7
*Source of condoms*					
Shop/drug store	7	5	12	16	75
Clinic	2	2	4	16	25
Ever been pregnant	0	2	2	19	10.5

**Note:** Totals do not always round to 100% because participants did not always respond to all the questions;

* Only those who responded yes were to answer the following questions

### Sexual reproductive health knowledge and behavior

#### Counseling services

Of a total of 137 respondents, 27 (19.7%) had ever gone to the hospital to seek advice on sex related issues (11 males, 16 females). Fifty seven (41.3%) out of 138 said they know where to go when they want to talk about sex (23 males, 34 females); of which 36 (63.1%) mentioned counselors/health care workers at the clinic (17 males, 19 females) and 19 (33.3%) mentioned friends, parents, and relatives (6 males, 13 females). A total 73 (55.3%) out of 132 respondents said they are able to discuss or get advice from parents/caregivers on sexual issues.

#### Family planning

**Figure 1:**
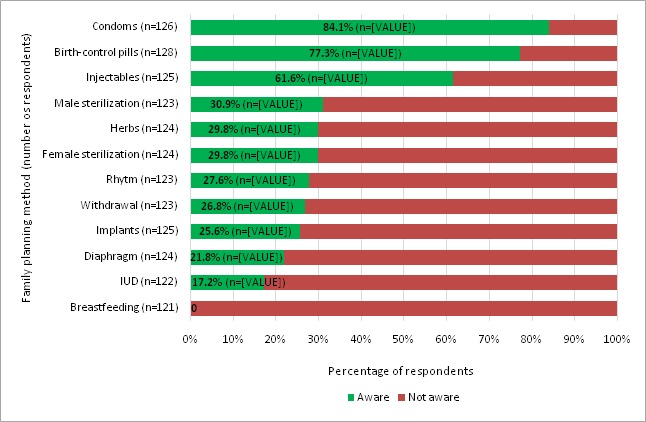
Awareness and use of family planning methods among HIV positive adolescents

Only 63 (47.0%) of 134 respondents had knowledge on ways to delay pregnancy; of these 43 (68.2%) were female. Among those who knew ways to prevent pregnancy, condoms were mentioned 26 times, followed by birth-control pills (13 times), abstinence (10 times), and withdrawal (2 times). When specifically asked about awareness and having used family planning (FP) methods (Figure 1), the most common FP methods which respondents were aware of, were again condoms, 84.1% of respondents (n=126) and birth-control pills 77.3% (n=128), followed by injectables, 61.6% (n=125); with 12,2 and 3 respondents who ever used these 3 methods respectively; any other mention of a method ever used was by 1 person for diaphragm. The majority of respondents were mostly unaware of other methods (69.1% to 100%), such as diaphragm, sterilization, and implants. A total of 76 out of all 148 participants (51.3%) were able to give at least one benefit of delaying pregnancy. Out of sexually active adolescents who use condoms and indicated source (n=14), the majority 10 (71.4%) accessed them from shops/drug stores while 4 (28.6% accessed them from the clinic.

#### Sexually transmitted infections

**Figure 2:**
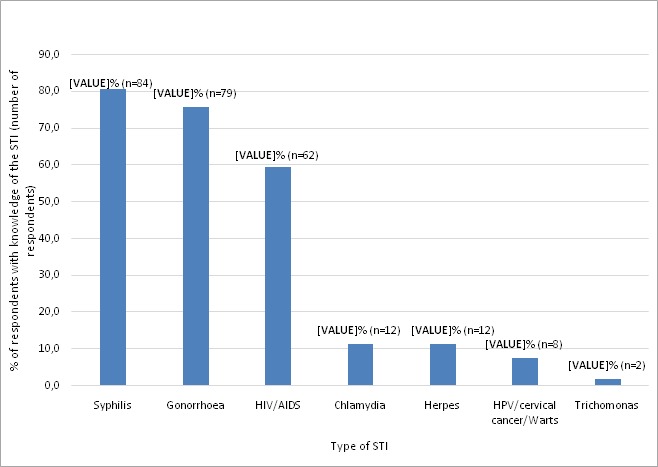
Distribution of percentages of HIV positive respondent adolescents with knowledge of different STIs

The majority 130 (92.2%) of 141 respondents indicated knowing about infections or disease that can be transmitted through sexual intercourse. As to whether they could list STIs they know, 105 (70.9%) responded yes, and of these 104 (99.0%) were able to cite at least one STI; 84 (80.8%) listed syphilis, 79 (76.0%) listed gonorrhea, and 62 (59.6%) listed HIV/AIDS, while fewer respondents listed other STIs (Figure 2). Regarding knowledge about STI symptoms, 65 (43.9%) responded; of these 56 (86.15) listed at least one symptom correctly. Out of 129 respondents, 86 (66.7%) said they knew how STIs are acquired, and 79 (91.9%) of them correctly stated how. Condoms was the most frequent method mentioned to prevent STIs (59.1%), followed by abstinence (53.9%) out of 115 respondents (Table 3). On believed dangers of not treating a STI, 86 (58.1%) participants responded; most of them mentioned death (67.4%), transmission to others (10.5%) and infertility (7.0%). The majority 87 (71.3%) of the 122 respondents were willing to inform their partner if they had a STI (Table 3); the main reasons being: it is the right thing to do, so their partner will get tested and treated, or so as not to infect partner. The remaining 35 (28.7%) were either unsure (14.7%), or wouldn’t want to inform their partner (13.9%). Some of the reasons for being unsure or not willing to inform the partner were: “because she would either think she gave me the STI or I have been cheating on her”; “because I wouldn´t want him to leave me”; “because she will think I´m unfaithful”; “Because I may not know the response or reaction to it”.

**Table 3:** Knowledge and beliefs about STIs among 15-19 years HIV positive adolescents

Knowledge and beliefs about STI	Yes	Respondents	%
Aware of STIs	130	148	87.8
Can cite an STI	104	148	70.3
Knows how STIs are transmitted	86	129	66.7
Can mention an STI symptom	65	148	43.9
Knows STI prevention method	115	148	77.7
Use of condom	68	115	59.1
Abstinence	62	115	53.9
Believes danger of not treating STIs is:			
Death	58	86	67.4
Transmission to others	9	86	10.5
Infertility	6	86	7
Would inform partner if have STI	87	122	71.3

**Figure 3:**
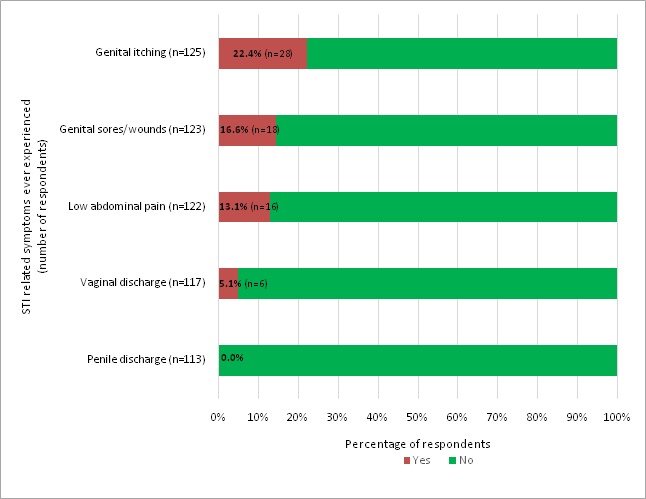
Frequencies of STI related symptoms ever experienced among HIV positive adolescents

When given a list of STI related symptoms to select those they ever experienced, 28 (22.4%) of the 125 respondents reported itching in their genitals, 14.6% mentioned having sores/wounds on their genitals, 13.1% reported low abdominal pain, and 5.1% indicated vaginal discharge. Overall, 42 out of 127 respondents had ever suffered from at least one STI related symptom (Figure 3 and Table 4).

**Table 4:** STI related symptoms ever experienced by 15-19 years old HIV positive adolescents

STI Symptoms ever experienced	Yes		No		No response	
	Male	Female	Male	Female	Male	Female
Penile discharge	0	-	41	-	13	-
Vaginal discharge	-	6	-	70	-	18
Sores/wounds on genitals	4	14	39	66	11	14
Itching on genitals	8	20	36	61	10	13
Low abdominal pain	4	12	40	66	10	16

### Determinants of sexual and reproductive health behaviors

While 41.3% of adolescents knew where to go to obtain information about sex, only 19.7% had ever gone to a doctor or health clinic to get information on sex. Adolescents currently not in school were over two times more likely to know where to go to talk about sex (OR= 2.53; 95% CI:1.10-5.82; p=0.02), and over 2 times more likely to have actually gone to seek advice on sexual issues (OR=2.61; 95% CI:1.04-6.58; p=0.03). Not attending school was also a significant predictor of knowing about the PCOE STI service as opposed to going to a private clinic (OR=2.7; 95% CI:1.03-7.41; p=0.03). Belonging to a peer support group was positive predictor of knowing where to go and seeking advice on sexual issues (OR=2.78; 95% CI:1.06-7.27; p=0.03). There was also a significant correlation between ever gone to seek counseling on sex and knowing ways to delay pregnancy (OR=2.60; 95% CI:1.06-6.41; p=0.03).

## Discussion

This survey among HIV infected adolescents 15-19 years showed that the majority had sexual experience; they have sexual desires and needs; some were in friendships; and most want to have healthy children. In a 2010 study among Zambian ALHIV, about half of older adolescents were in sexual relationships; some were married and some planned to have children in future [27]. This is consistent with a study on SRH needs of 15-19 year adolescents perinatally infected with HIV in Uganda [15]. In this study, 15.1% of the adolescents had ever had sex, lower than another Zambia study on ART and family planning among ALHIV where 21% of them had ever had sex (25% male and 75% female) [27]. These rates among ALHIV are much lower than among the general adolescent population in 2005 (44%) and 2009 (35%) [21]. Another 2001 Zambian study reported even higher rates of 62.1% and 48.7% among 15-19 male and female respectively [28]. Overall, the rate of sexual experience is lower among ALHIV compared to the general adolescent population in Zambia; suggesting both here and in another study that, because of their status, HIV positive adolescents may be delaying sexual debut [29]. While more girls than boys among the general Zambia adolescent population reported ever having had sex in the 2009 Zambia Sexual and Behavorial Survey [21] and in other studies among ALHIV in Zambia [19, 27], the current study revealed a reversal, with slightly lower rate among girls than boys.

In this study, fewer HIV positive adolescents had their first sex before the age of 15 years compared to the same age range in the general population of adolescents in Zambia [20] and among adolescents in Uganda where 14 percent of young women and men had their first sexual experience before they turn 15 years old [30]. Condoms and birth-control pills were the most known FP methods among the participants. Overall, 61.1% of sexually active respondents reported using condoms consistently, and only 61.9% said they had free access. This is lower than the 74% condom use among a similar adolescent population in Uganda [12]. The 61.1% rate of consistent condom use in this study correlates with the 61.9% who indicated use in the last sexual intercourse; this finding provides further confirmation as found elsewhere [31] that condom use at last coitus is a valid proxy for condom use behaviors spanning longer time periods. Slightly more adolescent females aged 15-19 (31%) than males (27%) reported using a condom at first sex [24]. About a quarter of the adolescents in this study were not aware of their HIV serostatus by the age of 14. The WHO recommends a gradual age-appropriate counselling and disclosure process for children up to 12 years according to their development stage [32]. Different models have been proposed [33–35] and different experiences reported from multiple countries [36–41]. A prospective study reported significantly decreasing age at disclosure over time [42]. In another Zambian study, only 37.8 % of children aged 8 to 17 years knew their HIV status [43], while a much lower proportion (17.4%) was observed in Ethiopia [44].

These findings suggest a need for interventions aimed at improving HIV disclosure to infected children through as this has been shown to have direct impact on treatment adherence [34]. Over half of the youth hadn’t disclosed their HIV status; similar findings were obtained in another study in Zambia [45] and in Thailand [39]. However, a Ugandan study reported only 2 out of 27 who hadn’t disclosed their HIV status to their sexual partner mainly for fear that it could end their relationships [27]. Delaying disclosure has been shown to adversely affect treatment adherence and overall psychological well-being and may also affect family functioning, adolescent social and academic life [37, 46, 47]. Not currently being in school was a predictor of knowing where to go and talk about sex, and also to have sought advice on sexual issues. This may be because they don’t have school commitments that prevents them from seeking and accessing sexual information. In a South Africa study, HIV knowledge and communication about sex was similarly distributed among students and non-students [48]. Being part of a peer support group was associated with knowing where to go for, and seeking advice on sexual issues; a similar finding from Zimbabwe suggested that children and careers perceived support groups as a safe social space for learning and acquiring information about HIV [49]. Some limitations of this study are that data was collected for adolescents at the PCOE facility. SRH topics are sensitive in general, and the participants didn’t always answer all the questions; this might have introduced some bias. The participants’ responses were based on their understanding and perceptions, and may not be extrapolated beyond the particular study context. The findings may not be generalized to the general HIV infected adolescent population because the study site was selected conveniently to accommodate time and financial constraints; although this is currently the only center where most adolescents are sent from Lusaka but also from across the country.

## Conclusion

This survey among HIV infected adolescents attending the PCOE-UTH provided insights into how they experience their sexuality, their sexual behavior and their SRH needs. The majority of HIV infected adolescents 15-19 years have sexual desire. Some are in romantic friendships, already engaged in sexual activities. They also express the desire to have healthy children. These adolescents are having to deal with their HIV and also the challenges associated with going from adolescence to adulthood. HIV infected adolescent SRH needs remain similar to those of the general population of the same age in terms of counseling on sexual matters, family planning and STI services. More efforts are needed to provide for adolescent health care needs and to create environments, such as one-stop centers, that are more aware, responsive, and tolerant of adolescent sexuality, especially for those living with HIV.

### What is known about this topic

- The number of adolescents living with HIV is continually increasing;
- Adolescents living with HIV are exploring their sexuality, are having sexual desire and are becoming sexually active;
- Adolescent living with HIV have needs for sexual reproductive health services.

### What this study adds

- More insight into the sexuality and sexual behaviors of adolescents living with HIV, as they remain sexually active, and being more knowledgeable about SRH;
- Not being in school appears to be a significant predictor for knowing where to go and seek for sexual related information and to actually have gone to access it;
- Compared to previous studies, there has been a gradual sexual debut delay over time among adolescents living with HIV in Zambia, however there are still gaps in meeting their SRH needs, in terms of health information, STI and family planning services.
